# Exploring on the Sensitivity Changes of the *LC* Resonance Magnetic Sensors Affected by Superposed Ringing Signals

**DOI:** 10.3390/s18051335

**Published:** 2018-04-25

**Authors:** Tingting Lin, Kun Zhou, Sijia Yu, Pengfei Wang, Ling Wan, Jing Zhao

**Affiliations:** 1 College of Instrumentation and Electrical Engineering, Jilin University, Changchun 130061, China; ttlin@jlu.edu.cn (T.L.); zhoukun16@mails.jlu.edu.cn (K.Z.); yusj6514@mails.jlu.edu.cn (S.Y.); wangpf6514@mails.jlu.edu.cn (P.W.); wanling@jlu.edu.cn (L.W.); 2 Key Laboratory of Geo-Exploration Instruments, Ministry of Education of China, Changchun 130061, China

**Keywords:** *LC* resonance magnetic sensors, ringing signals, noise level, detection sensitivity

## Abstract

*LC* resonance magnetic sensors are widely used in low-field nuclear magnetic resonance (LF-NMR) and surface nuclear magnetic resonance (SNMR) due to their high sensitivity, low cost and simple design. In magnetically shielded rooms, *LC* resonance magnetic sensors can exhibit sensitivities at the fT/√Hz level in the kHz range. However, since the equivalent magnetic field noise of this type of sensor is greatly affected by the environment, weak signals are often submerged in practical applications, resulting in relatively low signal-to-noise ratios (SNRs). To determine why noise increases in unshielded environments, we analysed the noise levels of an *LC* resonance magnetic sensor (*L* ≠ 0) and a Hall sensor (*L* ≈ 0) in different environments. The experiments and simulations indicated that the superposed ringing of the *LC* resonance magnetic sensors led to the observed increase in white noise level caused by environmental interference. Nevertheless, ringing is an inherent characteristic of *LC* resonance magnetic sensors. It cannot be eliminated when environmental interference exists. In response to this problem, we proposed a method that uses matching resistors with various values to adjust the quality factor *Q* of the *LC* resonance magnetic sensor in different measurement environments to obtain the best sensitivity. The LF-NMR experiment in the laboratory showed that the SNR is improved significantly when the *LC* resonance magnetic sensor with the best sensitivity is selected for signal acquisition in the light of the test environment. (When the matching resistance is 10 kΩ, the SNR is 3.46 times that of 510 Ω). This study improves *LC* resonance magnetic sensors for nuclear magnetic resonance (NMR) detection in a variety of environments.

## 1. Introduction

Nuclear magnetic resonance (NMR) technology is widely used in many fields such as biology, chemistry, physics, materials and geophysics [1,2]. Compared with high-field (HF) NMR, NMR at low-field *B*_m_ (LF) with Larmor frequency *f*_L_ in the kHz range offers some advantages [3]. For lower fields, i.e., surface nuclear magnetic resonance (SNMR) fields, the high homogeneity of the Earth’s magnetic field (50–60 μT) increases the amplitude and duration of the free induction decay (FID) signal [4]. In addition, the spin-lattice relaxation time T1 is more material dependent than in high field cases, resulting in improved T1-contrast imaging [5]. However, the disadvantage that accompanies the low-field is a weak signal. An effective method to improve the signal-to-noise ratios (SNRs) of LF-NMR measurements is to increase the sensitivity of pick up sensors. Because superconducting quantum interference devices (SQUIDs) have the highest sensitivities, they have been employed in LF-NMR measurements. The field resolutions of liquid-nitrogen-cooled (high-*T*c) SQUIDs and liquid-helium-cooled (low-*T*c) SQUIDs reach 30~50 fT/√Hz and 1 fT/√Hz respectively [6,7,8,9]. Recently, low-*T*c SQUIDs have also been used for SNMR detection [10]. Although SQUIDs yield the best field sensitivity, they are not commonly applied because of their susceptibility to flux trapping, their need for cooling to liquid He temperatures, and the operation difficulty. Hence, it is important to explore a more robust and sensitive sensor for practical use.

*LC* resonance magnetic sensors, also called receiving antennae, have been used for LF-NMR as well as SNMR systems to detect free induction decay (FID) signals of ground water due to their high sensitivity, low fabrication complexity, robustness and cost effectiveness [11,12,13]. Due to their weak signals, e.g., only nanovolts (10^−9^ V) in SNMR detections, large numbers of optimizations and designs have been conceived to increase SNR for *LC* resonance magnetic sensors [14,15,16]. In general, these improvements were mainly achieved by optimizing the coil physical characters and designing an *LC* resonance circuit filter network. As the key component of *LC* resonance magnetic sensors, different multi turn copper coils have been designed and sensitivity optimization was also studied by Lin et al. The high-sensitivity cooled coil system (77 K) developed in Lin’s study when matched with a low-noise preamplifier exhibits a sensitivity of approximately 2 fT/√Hz in a magnetically shielded room, which is comparable to that of low-*T*c SQUIDs [14]. In addition, Lin et al. improved the ability of an *LC* resonance sensor to detect weak signals by changing the coil’s operating temperature and optimizing the metre-level coils and used it for disaster water detection in a small underground space [15]. To further improve SNR, Zhang et al. designed a matching network consisting of an *LC* broadband filter in parallel with a capacitor, the capacitor was selected to increase the gain in the passband of the filter and improve the sensitivity of the sensor system [16].

At present, the disclosed method increases the SNR of the detection signal to some extent by changing the physical quantity of the sensor material and the temperature of the sensor application. However, they ignored an interesting phenomenon whereby the signal quality collected by the *LC* resonance magnetic sensor significantly differs in a magnetically shielded room and in the field. Furthermore, no one has fundamentally analysed the mechanism of the noise increase of *LC* resonance magnetic sensors in field environments or proposed corresponding solutions for that mechanism to optimize SNR.

In this study, we designed an *LC* resonance magnetic sensor and optimized it based on the mechanism whereby the sensitivity of *LC* resonance magnetic sensors depends on the environment. First, we designed an *LC* resonance magnetic sensor and compared the performances of a Hall sensor (*L* ≈ 0) with *LC* resonance magnetic sensor (*L* ≠ 0) in different environments. The ringing phenomenon and the question of whether the sensitivity of an *LC* resonance magnetic sensor (*L* ≠ 0) depends on its working environment will be addressed through experiments and simulations. Proof will be given that the superposition ringing of the *LC* resonance magnetic sensor caused by environmental interference is the reason for the increase in noise level. To solve this problem, the mechanisms of the decreasing sensitivity of *LC* resonance magnetic sensors should receive attention. By using matching resistors with various values to adjust the quality factor *Q* of the *LC* resonance magnetic sensor, optimum sensitivity can be obtained in different measurement environments. Finally, a LF-NMR experiment was used to verify the effectiveness of the method. This optimized design can effectively improve the SNR of the system by adjusting sensitivity according to the actual environment.

## 2. Materials and Methods

### 2.1. Device Selection of the LC Resonance Magnetic Sensor

**Figure 1:**
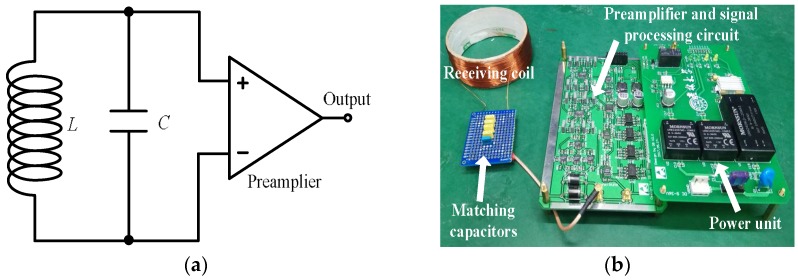
*LC* resonance magnetic sensor. (**a**) Schematic diagram; (**b**) sensor prototype.

The *LC* resonance magnetic sensor with non-negligible inductance *L* ≠ 0 is based on Faraday’s law of induction [17,18]. It is comprised of a receiving coil (*L*), a matching capacitor (*C*) and a preamplifier (shown in Figure 1). The receiving coil is connected to the matching capacitor in parallel to form a resonant circuit for improving the *Q* value, thus increasing the sensor’s sensitivity and suppressing the environmental noise outside the frequency range. In this work, an enamelled copper wire with a diameter of 0.4 mm was selected to fabricate the coil of the magnetic sensor. The layers, number of turns and diameter of the coil were 3, 147 and 80 mm, respectively. The coil’s direct current resistance was 1.73 Ω (@300 K), and the measured inductance was approximately 1.68 mH. We used 2.7 µF capacitors to match the coil to achieve a resonance frequency *f*_0_ of 2362 Hz with *Q* = 10.3. An AD797 operational amplifier produced by Analogue Devices, USA connected to the *LC* resonance circuit was employed as a preamplifier with a voltage noise of 0.9 nV/√Hz and current noise of 2 pA/√Hz in the white noise region.

### 2.2. Sensitivity Analysis of the LC Resonance Magnetic Sensor

The intrinsic voltage noise of the *LC* resonance magnetic sensor consists of three parts [14]: the thermal noise of the coil $V_{T}$, the preamplifier’s voltage noise $V_{n}$ and current noise $I_{n}$. The sensor’s voltage noise $V_{S}$ is described as

(1) $$V_{S}=\sqrt{V_{n}^{2}+{\left(I_{n}^{2}Z\right)}^{2}+{\left(Q\sqrt{4k_{B}TR_{S}}\right)}^{2}}$$

where *Q* denotes the quality factor of the *LC* resonance circuit, *Z* denotes the impedance at the resonance frequency, $k_{B}$ is Boltzmann’s constant, *T* is the coil temperature, and $R_{S}$ is the direct current resistance of the coil. According to Equation (1), the calculated voltage noise of the developed *LC* resonance magnetic sensor is approximately 2 nV/√Hz@300 K.

According to Faraday’s law of induction, the transfer coefficient of the sensor can be described as

(2) ∂V/∂B=QNSω

where *N* denotes the number of coil turns, *S* is the average detection area, and $\omega =2\pi f_{0}$ is the resonance angular frequency.

By combining Equations (1) and (2), we can obtain the sensitivity of the *LC* resonance magnetic sensor, which is expressed as

(3) $$B_{n}=\frac{V_{S}}{\left(\partial V/\partial B\right)}=\frac{\sqrt{V_{n}^{2}+{\left(I_{n}^{2}Z\right)}^{2}+{\left(Q\sqrt{4k_{B}TR_{S}}\right)}^{2}}}{QNS\omega }$$

To obtain the optimal detection sensitivity, the structure parameters of coil and the performance of the preamplifier should be considered.

### 2.3. Manual Interference Sensor Noise Test Method

The noise of the *LC* resonance magnetic sensor with manual interference was tested in a shielded room. A transmitter coil was placed 5 cm above the *LC* resonance magnetic sensor coaxially, and a signal generator was used to output the fixed amplitude and frequency waveforms on the transmitting antenna to generate manual interference. At that point, the *LC* resonance magnetic sensor was used to collect noise, and the data was finally displayed and saved by the dynamic signal analyser (Agilent 35670A produced by Agilent Technologies, Santa Clara, CA, USA). By setting different amplitudes and different frequencies of the manual interference sources, the influence of environmental interference on the sensitivity of the *LC* resonance magnetic sensors was explored. The detailed configuration of this test method is provided with the results of the manual interference noise test.

### 2.4. LF-NMR Experimental Method

The LF-NMR experiment was conducted in a magnetically shielded room. In the experiment, 3 L of water was used as the sample to be tested and was placed in the centre of the static magnetic field *B*_m_ generated by the Helmholtz coil. The excitation magnetic field *B*_p_ generated by the launch module was used to excite the target water body. An FID signal acquisition module comprised of an *LC* resonance magnetic sensor and a signal conditioning circuit was used for signal acquisition and preliminary processing. The FID signal was finally displayed and stored by the data acquisition module. In this experimental environment, by changing the resistance of the matching resistor *R*_m_, the *LC* resonance magnetic sensor collected noise and FID signals at different sensitivities and ultimately analysed the quality of the acquired signal. The configuration details of this experiment are provided together in the results.

## 3. Results

### 3.1. The Voltage Noise Comparation with or without Environmental Interference

**Figure 2:**
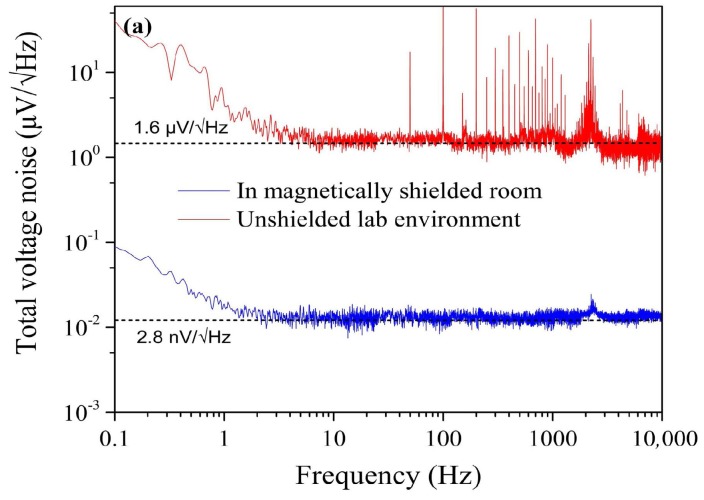

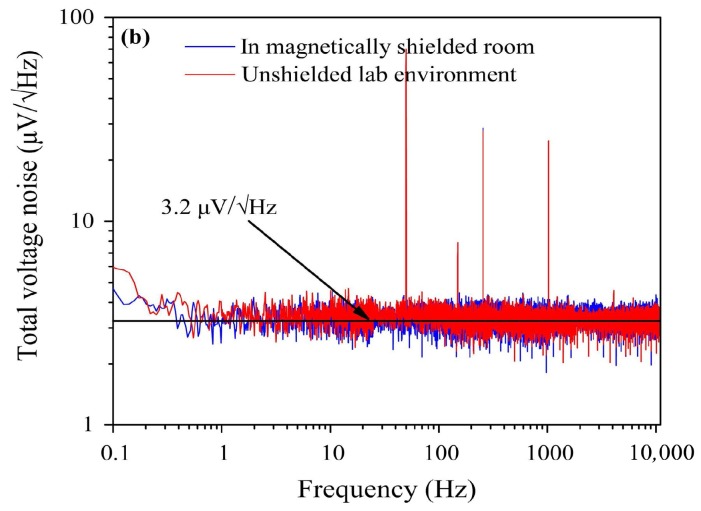
Comparison of the measured voltage noise in unshielded lab environment and in a magnetically shielded room. The blue curve represents the detection in magnetically shielded room and the red curve represents the detection in unshielded lab environment. (**a**) The *LC* resonance circuit with *L* ≠ 0; (**b**) The Hall sensor with *L* ≈ 0. All noise spectra in this paper are recorded by a dynamic signal analyzer (Agilent 35670A produced by Agilent Technologies, Santa Clara, CA, USA).

To compare the voltage noise of the *LC* resonance magnetic sensor in different environments, we placed it in a laboratory environment with abundant power line interference and in a magnetically shielded room, with shielding factors of 68 dB@10 Hz and 80 dB@100 Hz, respectively. The results are shown in Figure 2a. The measured white noise level of the sensor in the magnetically shielded room was 2.8 nV/√Hz, as shown by the blue curve, which was close to the above-calculated value of 2 nV/√Hz. In contrast, the white noise (>5 Hz) reached up to 1.6 µV/√Hz in the lab environment, although there was no interference below 50 Hz, as shown by the red curve. Here, many power line peaks (50 Hz and its harmonics) were clearly observed. An interesting phenomenon is that the whole noise level increased 3 orders of magnitude due to the interference.

For comparative analysis, a sensor was selected with a negligible inductance *L* ≈ 0, i.e., a Hall sensor [19,20]. The Hall sensor’s white noise remained unchanged at 3.2 µV/√Hz both in the lab environment and in the magnetically shielded room, as shown in Figure 2b. Here, the SS495A Hall effect sensor produced by Honeywell Solid State Electronics Center was used. From Figure 2, we learned that the noise behaviour of the *LC* resonance magnetic sensor with *L* ≠ 0 differed in different environments, whereas that of the Hall sensor with *L* ≈ 0 did not.

### 3.2. The Noise Test and Analysis of the Sensor with Manual Interference

Generally, there are two types of interference in a typical laboratory environment: square-wave-shaped disturbances from digital instruments and sinusoidal waves of different frequencies (i.e., power line interference and its harmonics).

**Figure 3:**
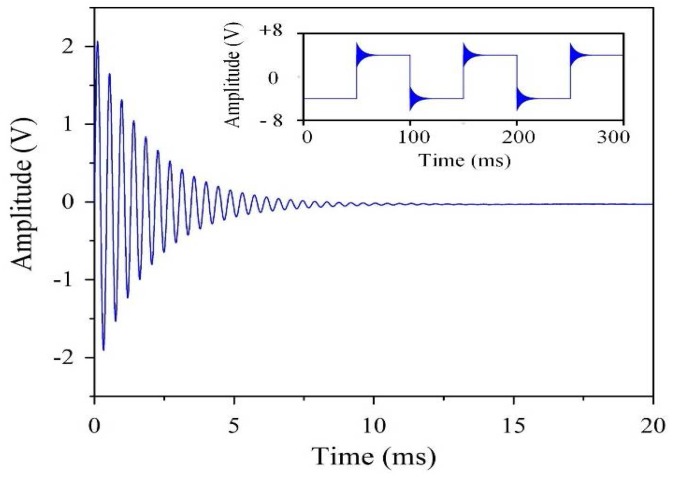
Ringing signal recorded by the *LC* resonance magnetic sensor. The inset shows the ringing appearing after each rising and falling edge of the square wave.

To study the impact of the environmental interference on the sensitivity of the *LC* resonance magnetic sensor, we placed it in the magnetically shielded room again and applied some manual interference. One single turn coil with a diameter of 80 mm was employed as the transmitting antenna to generate the manual interference. The antenna was coaxially placed next to the *LC* resonance magnetic sensor at a distance of 5 cm. A function generator yielded a square wave with an amplitude of 60 mA and a frequency of 10 Hz to the transmitting antenna. A transient ringing signal was clearly detected, as shown in Figure 3. The inset reveals that the ringing signal appeared after each rising or falling edge of the square wave. The 2362 Hz ringing frequency was the intrinsic resonant frequency *f*_0_. It exponentially decayed from an initial amplitude of ±2.1 V (here, the amplifier gain = 100). After approximately 15 ms, it submerged below the system noise. The measurable ringing duration was determined by its initial amplitude [21,22]. A ringing time constant of $\tau_{r}=2Q/\omega =1.39$ ms was observed. The duration of the ringing signal could exceed 10 $\tau_{r}$ in this case. The initial ringing amplitude increased with the increasing amplitude of the applied square wave.

**Figure 4:**
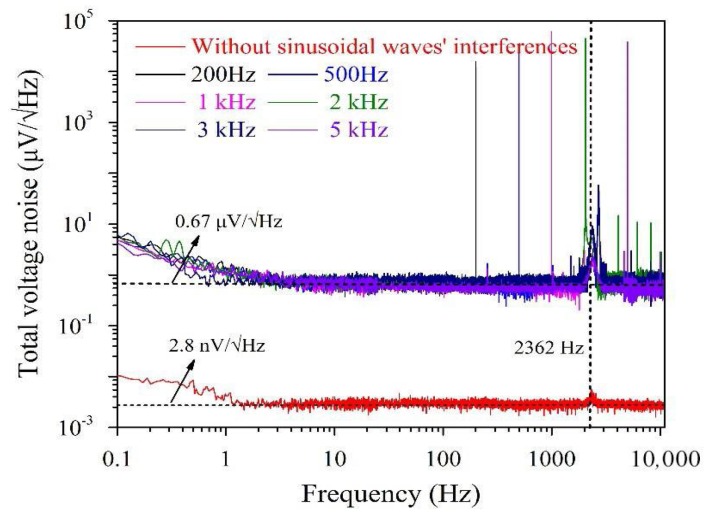
The spectra of the *LC* resonance magnetic sensor with and without manual sinusoidal waves’ interference in a magnetically shielded room.

We also applied sinusoidal waves with amplitudes of 20 mA and frequencies of 200 Hz, 500 Hz, 1 kHz, 2 kHz, 3 kHz and 5 kHz to the transmitting antenna to generate a manual interference of 493 pT in the centre. The measured noise spectra are shown in Figure 4. With the exception of some spectral peaks that were generated by manual interference, the noise figures remained almost constant. However, the noise level increased up to 0.67 µV/√Hz, which was nearly 240 times larger than that without interference.

**Figure 5:**
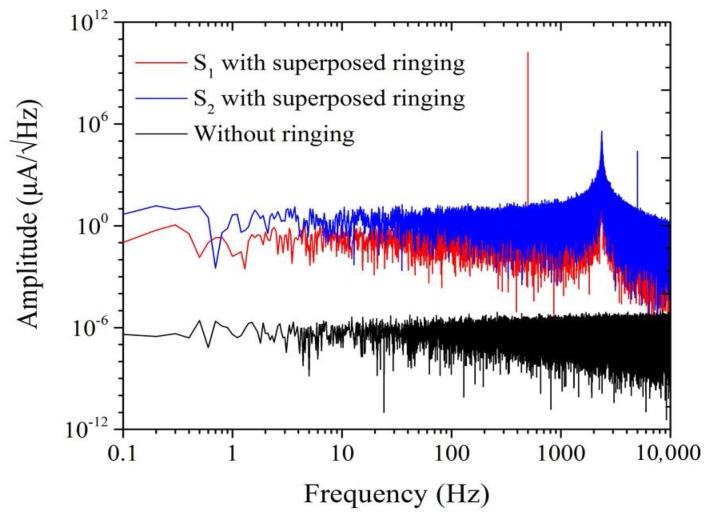
The simulated noise level with and without ringing.

To understand the mechanism of the white noise increase due to the ringing effect, we performed a numerical simulation using MATLAB software with 2016 version. White noise with a mean value of 0 and standard deviation of 0.2 µA was randomly added to the *LC* resonance magnetic sensor. The sampling rate and recording time were set to 50 kHz and 10 s respectively. Its power spectrum density is shown by the black line in Figure 5. Sinusoidal (S) waves with different amplitudes (*A*) and frequencies (*f*) were superposed with the above mentioned random noise, i.e., wave S_1_ with *A* = 0.1 A and *f* = 500 Hz and wave S_2_ with *A* = 100 µA and *f* = 5 kHz. For both superimposed signals, at each point where the sinusoidal waves passed zero, one ringing with a frequency of 2362 Hz was added to the superimposed signals. The amplitudes of the ringing signals were chosen randomly from 100 to 1000 µA, and the phase was switched between 0 and π. The spectra of the superimposed signals are shown by the blue and red lines in Figure 5. As expected, the white noise levels for both of the sinusoidal waves increased by approximately five orders of magnitude, which can be explained by the superimposed ringing.

Due to *L* ≈ 0, the Hall sensor and SQUID did not present the ringing effect. Therefore, their sensitivities remained unchanged in the distributed environments. The environmental interferences were the primary reason for the reduction in the detection sensitivity of the *LC* resonance magnetic sensor. To achieve optimal sensitivity for applications in ambient environments, adjusting the *Q* of the *LC* resonance magnetic sensor by connecting a resistor to the matching capacitor in parallel is an effective method.

### 3.3. LF-NMR Experiment using the LC Resonance Magnetic Sensor

**Figure 6:**
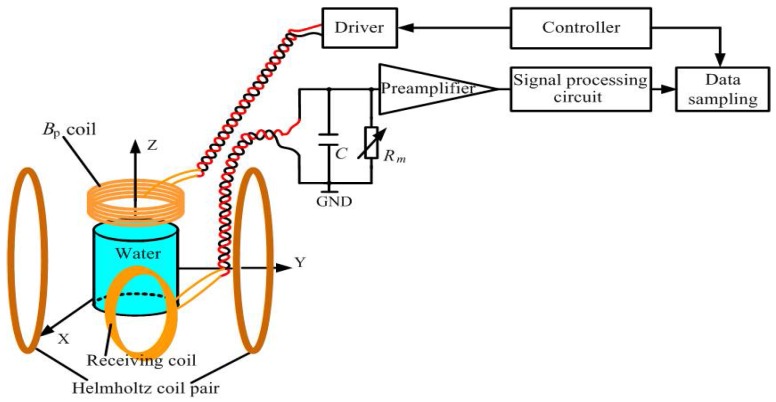
Schematic Diagram of LF-NMR Experimental Model.

We connected a resistor (*R*_m_) to the matching capacitor in parallel to change the *Q* value of the *LC* resonance magnetic sensor to explore its ability to suppress noise with different sensitivities for detecting an actual water signal. A simple LF-NMR experiment was carried out inside the magnetically shielded room. LF-NMR experimental models with different sensitivities are shown in Figure 6. They were mainly comprised of four parts. (i) A Helmholtz coil pair was used to generate a static magnetic field *B*_m_ = 54.78 μT, which determined the Larmor frequency *f_L_* = 2.333 kHz. The 3 L water sample was located in the centre of the Helmholtz coil; (ii) A *B*_p_ transmitting coil made of multi-turn enamelled wire with a diameter of 2.26 mm was placed 30 cm above the water sample to generate a 0.8 mT *B*_p_ pulse for polarizing the water samples. The driver was a controllable current source used to provide current to the *B*_p_ coil for 7 s; (iii) The FID signal acquisition unit included the *LC* resonance magnetic sensor (receiving coil *L*, matching capacitor C, matching resistor *R*_m_ and preamplifier) and signal processing circuit. The value of the matching capacitor was slightly modified for the *LC* resonance frequency corresponding to the Larmor frequency of the static magnetic field. After the *B*_p_ pulse was over, a release time of 30 ms passed before initiating collection of 500 ms signals. The signal processing circuit was a bandpass filter comprised of an eighth-order Butterworth low-pass filter and eighth-order Butterworth high-pass filter and had cut-off frequency of 1.5 kHz–3.7 kHz [15]; (iv) Regarding the experimental controller and data sampling, Altera’s EPM1270T144C5N Complex Programmable Logic Device (CPLD) was used to control the working status of each part. The data sampling was completed using a dynamic signal analyser (Agilent 35670A). The data for each experiment was saved after 30 superpositions to avoid influences due to chance factors. The sensing directions of the receiving coil, *B*_p_ and *B*_m,_ were perpendicular to each other.

**Figure 7:**
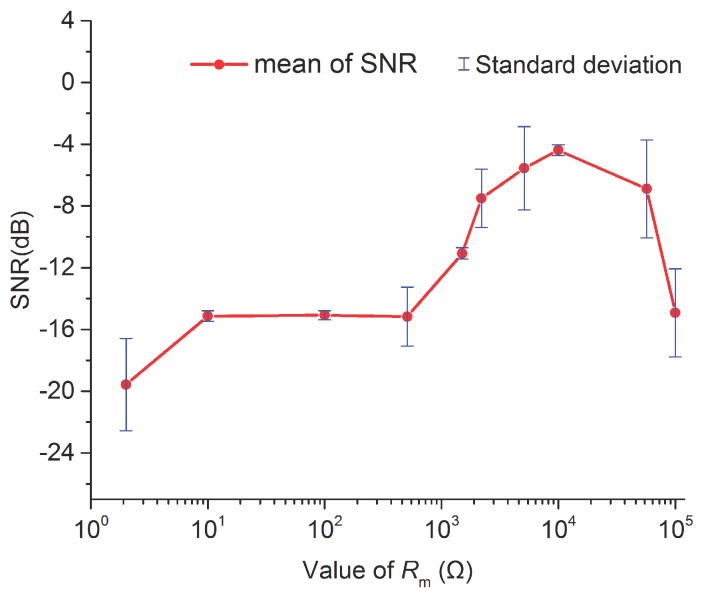
SNR of the FID signal with different value of *R*_m_; Red dots represent the mean of SNR and the blue lines represent the standard deviation.

As mentioned above, to explore the sensors’ ability to suppress noise with different sensitivities for detecting the actual water signal, we used a parallel resistance *R*_m_ to adjust the *Q* value. Through several experiments, we obtained the diagram between the SNR and *R*_m_ values shown in Figure 7, where SNR was calculated according to the effective signal and noise powers. The red dots represent the averages of three sets of parallel data, and the blue line represents standard deviation. It can be seen from the Figure 7 that for *R*_m_ below 1 kΩ, the SNR of the FID signal was generally lower and that with the continuous increase in *R*_m_, the SNR improved, but the error fluctuation was large. When *R*_m_ = 10 kΩ, the SNR of the FID signal reached a summit, and the deviation was minimal. As the value of *R*_m_ continued to increase, the SNR of the signal slightly decreased, but the deviation range greatly increased. Based on the above experimental results, we believe that noise suppression can be achieved to some extent by changing the value of the parallel resistance *R*_m_. Therefore, in an NMR actual application, the SNR can be improved by modifying the value of the parallel resistance of the *LC* resonance sensor in accordance with the noise levels of different detection areas.

**Figure 8:**
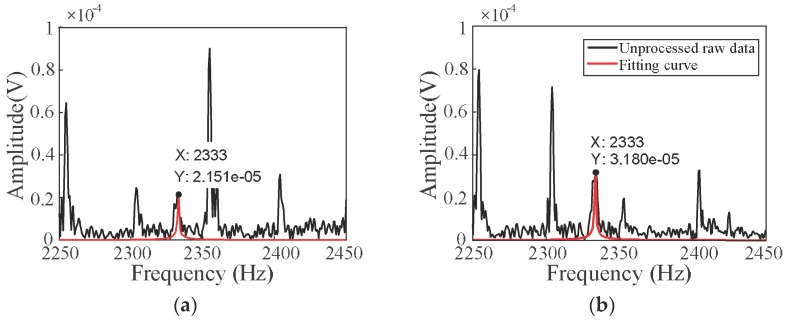
The spectrum of the FID signal; the black curve represents the detection data and the red curve represents the fitting data. (**a**) Spectrum of FID signal when *R*_m_ = 57 kΩ; (**b**) Spectrum of FID signal when *R*_m_ = 10 kΩ.

To further demonstrate that changing the matching resistor *R*_m_ can improve the SNR of the signal, we analysed the frequency spectrum of the FID signal by extracting two groups of data from the LF-NMR experiment with values of *R*_m_ = 57 kΩ and *R*_m_ = 10 kΩ, as shown in Figure 8. The *x*-axis and *y*-axis represents the frequency of the signal and the amplitude of the FID signal, respectively. The black lines represent the unprocessed raw data, and the red lines represent the fitting curve. The frequency of the FID signal was 2333 Hz, i.e., the peak of the red lines. By comparison, in addition to 50 Hz power frequency harmonics, the amplitude of the FID signal with *R*_m_ = 10 kΩ was obviously higher than that for *R*_m_ = 57 kΩ. Therefore, the experimental results further confirmed that choosing the right *LC* resonance magnetic sensor matching resistor value according to the measurement environment can achieve the best sensitivity and obtain high quality FID signals in NMR applications. The signal processing process was divided into two steps. First, a Fourier transform was performed on the collected time domain signal and then the fitting curve was marked, as shown by the red curve in Figure 8.

## 4. Discussion and Conclusions

In the present study, we compared the behaviours of magnetic sensors with *L* ≈ 0 and *L* ≠ 0 in a magnetically shielded room and in an unshielded field environment to observe the changes in the noise levels of different sensors. The results showed that the noise behaviours of the *LC* resonance magnetic sensor with *L* ≠ 0 differed in the various environments, whereas those of a Hall sensor with *L* ≈ 0 were constant. We then explored the ringing phenomenon and approved that the superposed ringing effects caused by environmental interference adjusted the detection sensitivity of the *LC* resonance magnetic sensors. In response to that problem, we proposed a method that uses matching resistors with various values to adjust the quality factor *Q* of the *LC* resonance magnetic sensor in different measurement environments to suppress the noise. Additionally, in the future a special design structure of the *LC* resonance magnetic sensor can be adopted to reduce the inductance *L*, thereby suppressing the noise levels of the sensors.

A LF-NMR experiment was used to verify the effectiveness of the method. We analysed the SNRs and frequency spectra from multiple test results, when the matching resistance was 10 kΩ, the SNR was 3.46 times than that of 510 Ω, and the amplitude of the FID signal with *R*_m_ = 10 kΩ was obviously higher than that of *R*_m_ = 57 kΩ in the test environment. On that basis, it was concluded that for different measurement environments, adjusting the matching resistor can improve the quality factor *Q* of the *LC* resonance magnetic sensor to obtain the best sensitivity, thus suppressing noise and improving SNR in NMR applications.
